# Proper detailed parameters for S1 sacral alar iliac screw placement in the Chinese population, a 3D imaging study

**DOI:** 10.1186/s13018-018-0739-8

**Published:** 2018-02-26

**Authors:** Yao Wang, Wenhao Hu, Fanqi Hu, Hao Zhang, Tianhao Wang, Yan Wang, Xuesong Zhang

**Affiliations:** Chinese PLA General Hospital, 28th Fuxing Road, Beijing, China

**Keywords:** Pelvic fixation, S1 alar-iliac screw, Anatomic study, Computed tomography reconstruction

## Abstract

**Background:**

S1-AI technique may be used as a salvage technique in pelvic fixation of complex spinal deformity surgery. However, the proper detailed parameters in the Chinese population has not been analyzed before to instruct S1-AI screws placement and to ensure the safety of clinical application while the trajectory in pelvic changes significantly at each angle.

**Results:**

The ideal S1AI screw trajectory could be obtained in 28 of 30 female patient images (93.3%) and in all of the male patient images (100%). The screws that have already been used clinically in S2AI pathways can be applied in S1AI fixations.

**Conclusion:**

It is feasible to place S1AI screws in 93.3% of female Chinese adult patients and in all male Chinese patients. Preoperative CT reconstruction should be performed to evaluate proper trajectory parameters and to avoid anterior violation.

## Background

Lumbar-sacral fusion has been utilized in many clinical scenarios such as flat-back syndrome and kyphosis, pelvic obliquity, high-grade spondylolisthesis, and extensive sacropelvic tumor resection [1–6]. However, screws in the first sacral vertebrae do not always hold up to the loads applied since S1 pedicles are capacious and often contain less-supportive cancellous bone. The S2 alar iliac (S2AI) technique was described to facilitate deformity correction [4, 7, 8], which simultaneously adding dissection of the skin, subcutaneous tissue, and muscle in this area that may increase the risk of blood loss and wound healing problems. To the best of our knowledge, in the Chinese population, the detailed proper parameters have not been analyzed before to ensure the safety of clinical application of S1 alar iliac (S1AI) screws while the ideal routeway changes significantly on different angle of the pelvic. The purpose of this study is to determine the anatomical feasibility and ideal trajectories of placing S1-AI screws in the Chinese population and to instruct clinical application.

## Methods

### Subjects

After approval from our institutional review board, 60 nonconsecutive computed tomography (CT) scans of the pelvis were randomly and retrospectively selected. The scans belonged to 60 Chinese skeletally mature patients were performed to investigate hypogastralgia (46 cases), urinary tract calculi (6 cases), or rectal tumor (8 cases). All patients scanned with bony diseases or deformity were excluded from this study. The pelvis were scanned by a 16-slice CT (UNITED IMAGING 16-slice CT scanner; uCT 510; China). Power settings were typically 120 kV and 220 mA, 800 ms rotation time with a slice thickness of 1.5 mm. The field of view was contained 512 × 512 pixels and increments of 1 mm, using detector collimation of 16 × 0.6 mm (pitch 0.9375). All of the CT scans were analyzed using UNITED IMAGING workplace Zheng He 61 (China, United Imaging Company), a matched CT imaging computer application for three-dimensional (3D) interactive viewing, manipulation and measurement of the anatomical structures, and all parameters with high accuracy. We used systematic random sampling for selecting every 15th patient from a list generated from our institution’s Picture Archiving and Communication Systems (PACS) of patients who had been scanned from the beginning of June 2016 to the end of October 2016. The initial case was randomly selected. Our study population was consisted of 60 patients (30 males and 30 females) with a mean age of 46.2 years (range 21–72 years).

### CT imaging manipulation and parameter measurements

**Fig. 1:**
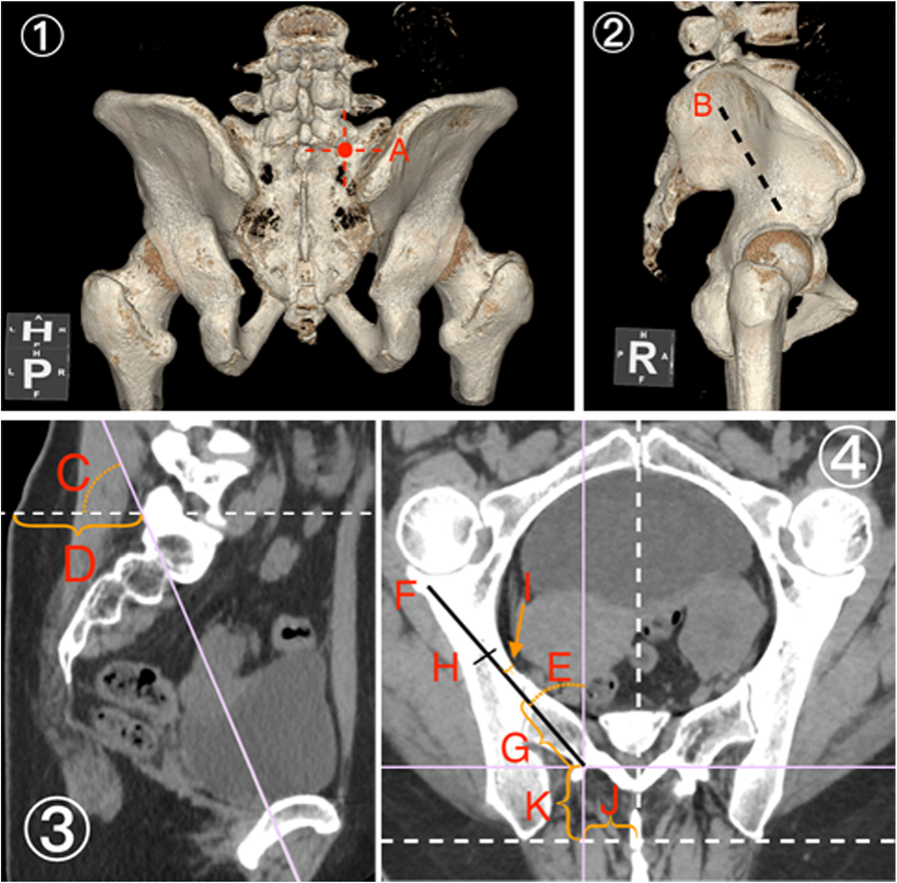
Case 7, 51-year-old female. **1** Posterior view of CT 3D imagine. A The entry point of S1AI trajectory. **2** Lateral view of CT 3D imagine. B The direction of S1AI trajectory. **3** Sagittal reconstructed plane of S1AI trajectory. C Sag. A.: 66.7°; D Skin D.: 63.8 mm. **4** Transverse plane of S1AI trajectory. E Tsv. A.: 40.3°; F Max. L.: 113.9 mm; G Sac. L.: 46.7 mm. H Iliac W.: 17.2 mm; I Cortex D.: 5.6 mm; J Mid. D.: 24.2 mm; K PSIS D.: 29.4 mm

The CT imaging manipulations were performed and rotated until they matched the ideal S1-AI trajectory (greatest length and width of osseous channel) for patients. The entry point of S1-AI screws was selected at the lateral sacral crest between the S-1 foramen and the margin of the S-1 superior endplate laterally according to previous studies [6, 9–11], which is also the traditional entry point of S1 pedicle screw (Fig. 1(1a)).

**Fig. 2:**
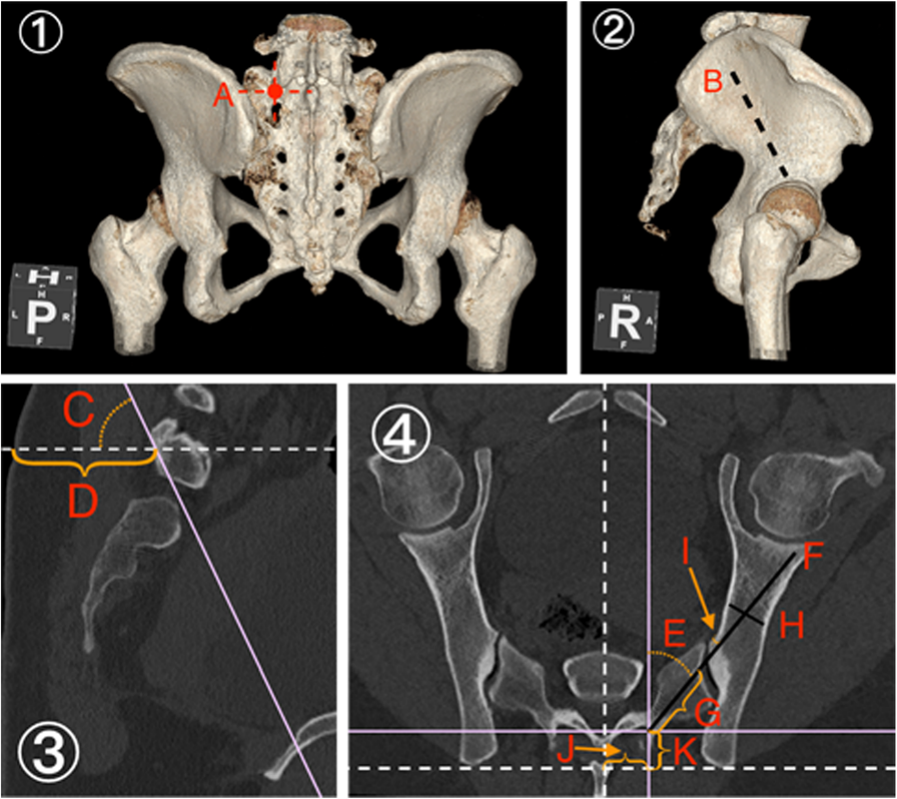
Case 2, 55-year-old female. **1** Posterior view of CT 3D imagine. A The entry point of S1AI trajectory. **2** Lateral view of CT 3D imagine. B The direction of S1AI trajectory. **4** Sagittal reconstructed plane of S1AI trajectory. C Sag. A: 62.0°; D Skin D.: 59.1 mm. **4** Transverse plane of S1AI trajectory. E Tsv. A.: 37.4°; F Max. L.: 121.1 mm; G Sac. L.: 43.1 mm. H Iliac W.: 14.5 mm; I Cortex D.: 2.0 mm; J Mid. D.: 24.7 mm; K PSIS D.: 19.8 mm

The detailed measurements within the determined trajectory were outlined as below (demonstrated in Figs. 1 and 2):

- Sag. A.: The caudal trajectory angulation in the sagittal plane (Fig. 1(3c)).
- Skin D.: The shortest distance from the starting point to the skin (Fig. 1(3d)).
- Tsv. A.: The lateral angulation in the transverse plane (Fig. 1(4e)).
- Max. L.: The maximal length of the trajectory from the starting point to the anterior cortex (Fig. 1(4f)).
- Sac. L.: The length of intrasacral trajectory (Fig. 1(4g)).
- Iliac W.: The narrowest iliac width between the inner cortices of the ilium in the transverse plane (Fig. 1(4h)).
- Cortex. D.: The narrowest distance between the trajectory and the nearest inner cortex surface (Fig. 1(4i)).
- Mid. D.: The distance of the starting point lateral from the middle line of sacrum (Fig. 1(4j)).
- PSIS D.: The distance from the starting point to the PSIS (Fig. 1(4k)).

### Statistical analysis

The results were represented as mean ± standard deviations for variables. To detect possible divergence of the parameters between genders, independent samples’ *t* test was performed in this study. Statistical significance was set at *p* < 0.05. All analyses were carried out using the SPSS (Statistical Package for the Social Sciences, version 17).

## Results

The parameters for the S1AI pathway were measured through the three-dimensional radiographic analysis (Table 1, Figs. 1 and 2).

**Table 1:** Parameters of S1-AI screw trajectory measurements (mean ± standard deviation)

	Males (*n* = 30)			Females (*n* = 30)		
Parameters	Left	Right	*p*	Left	Right	*p*
Sag. A. (°)	57.21 ± 5.24*	55.68 ± 5.75*	0.455	59.52 ± 6.27	59.41 ± 5.35	0.760
Skin D. (mm)	53.06 ± 9.09	53.71 ± 9.56	0.853	57.35 ± 7.45	57.11 ± 6.78	0.927
Tsv. A. (°)	37.01 ± 2.39	37.10 ± 2.77	0.929	36.49 ± 2.79	37.24 ± 3.56	0.538
Max. L. (mm)	120.96 ± 4.03*	119.27 ± 4.34*	0.281	113.25 ± 6.11	111.69 ± 6.22	0.496
Sac. L. (mm)	41.50 ± 5.18*	42.91 ± 4.38*	0.425	39.90 ± 7.10	41.42 ± 5.58	0.520
Iliac W. (mm)	21.64 ± 4.17*	21.83 ± 3.76*	0.897	16.83 ± 1.59	17.25 ± 1.12	0.411
Ctx D. (mm)	8.31 ± 1.87*	8.46 ± 2.16*	0.850	5.69 ± 1.27	5.78 ± 0.86	0.687
Mid. D. (mm)	27.50 ± 1.51*	28.04 ± 2.47*	0.486	26.20 ± 2.92	26.67 ± 2.41	0.532
PSIS D. (mm)	27.41 ± 4.24*	27.48 ± 3.89*	0.964	22.30 ± 5.73	22.39 ± 5.41	0.851

*Statistically significant if *p* < 0.05 compared with the data of females

Based on the ideal S1AI trajectories of males, the mean sagittal angle was 57.21 ± 5.24° on the left and 55.68 ± 5.75° on the right. The average transverse angle was 37.01 ± 2.39° for the left pelvis and 37.10 ± 2.77° for the right pelvis. Compared with the parameters above, the pathways of females were significantly more caudal with mean sagittal angle of 59.52 ± 6.27° on the left and 59.41 ± 5.35° on the right (*p* = 0.038). However, the transverse angle of females were almost the same with males (*p* = 0.797).

The maximal mean S1AI pathway of males was 120.96 ± 4.03 mm (range, 114.5–127.4 mm) for the left pelvis and 119.27 ± 4.34 mm (range, 113.9–129.1 mm) for the right pelvis, while the trajectories of females were significantly shorter (*p* < 0.001) with 113.25 ± 6.11 mm (range, 103.5–125.6 mm) on the left and 111.69 ± 6.22 mm (range, 102.2–121.6 mm). Based on the idealized trajectory for this technique, the pathway traversed a mean 41.50 ± 5.18 and 42.91 ± 4.38 mm of the sacral bone before crossing the anterior portion of the sacral alar into the ilium of males for the left and right pelvis, respectively, while 39.90 ± 7.10 and 41.42 ± 5.58 mm for the pelvis of females (*p* = 0.274). The narrowest mean width of the ilium of males along the chosen trajectory was 21.64 ± 4.17 mm for the left side and 21.83 ± 3.76 mm for the right. The mean width at the thinnest portion of the ilium of females was 16.83 ± 1.59 and 17.25 ± 1.12 mm, which were more narrower than that of males (*p* < 0.001).

With respect to the cortex distance (the narrowest distance between the trajectory and the nearest inner cortex surface), the parameters were 8.31 ± 1.87 mm (range, 6.3–13.1 mm) on the left and 8.46 ± 2.16 (range, 5.5–14.3 mm) on the right. As for the females, the parameters were 5.69 ± 1.27 mm (range, 2.0–7.2 mm) on the left and 5.78 ± 0.86 mm (range, 4.1–7.5 mm) on the right. In two of the 30 female cases (3.3%), we found that the cortex distance were under 3.75 mm, which were 2 and 2.2 mm.

Based on the ideal trajectory for the S1AI screw, the distance below the skin for S1AI screw insertion point showed no statistical differences between the males and females. The entry points of males away from the PSIS were 5 mm longer than those of females on both sides and approximately 1 mm longer away from the midline.

## Discussion

It is a challenging area that pelvic fixation continues to be in spine surgery. The biomechanical forces, anatomy, and bone quality are some reasons why spine surgeons continue to explore options for fixation in fusions for deformity that extend to the pelvis [12].

The technique for placement of S2AI screws was contemporaneously described by Dr. Sponseller and Dr. Kebaish in pediatric and adult populations, respectively [7, 13, 14]. According to previous studies, S2AI screw fixation technique improved construct stability and biomechanical torsion due to the direction and the longer length of the screws [15–17] and reduce complications including implant prominence due to the low profile of the screws [4, 18–20]. In 2013, Dr. Mattei et al. reported a technique involving the use of combined S-1 and S-2 SAI screws as a salvage procedure for definitive pelvic fixation after pseudarthrosis in the lumbosacropelvic junction [6]. Though the S1AI screw technique had already been used clinically [21], no anatomic parameters have been analyzed for proper trajectory of S1AI screw.

Our analysis illustrated that the traditional entry point of S1 pedicle screw can serve as a satisfied entry point of S1AI screw to achieve ideal implement trajectory. The maxlength of S1AI trajectories was 120.06 ± 4.21 mm (range, 113.9–129.1 mm) for the males and 112.47 ± 6.11 mm (range, 102.2–125.6 mm) for the females. Our assessment of the iliac width, which served to evaluate for the maximal anchor diameter, ranged from 17.4 to 32.4 mm for males and 13.5 to 20.3 mm for females within the determined S1AI trajectory. Therefore, a standard S2AI screw that ranges from 70 to 100 mm in length and 5.0–7.5 mm in diameter reported in previous studies [6, 7] can be appropriate to Chinese population for S1AI screw application. In our observation, the S1AI trajectories were heading directly to the acetabulum so that excessively long screws could damage the hip joint.

The minimum parameters of cortex distance for S1 ideal pathways demonstrated the risk of anterior violation. One S1AI trajectory of the 30 females (6.7%, 2 mm, S1L, Fig. 2) was not sufficient for currently available screws with a minimum diameter of 5.0 mm screw, which indicates the danger of impingement. Anterior violations, although occurring very infrequently, may have important deleterious consequences due to the injury of several important vascular, neurological, and visceral structures located inside the pelvis [22]. Therefore, preoperative CT scans and reconstruction should be performed to evaluate proper trajectory parameters and to choose appropriate screws.

According to the ideal trajectory direction, our study recommended insertion of the S1AI screws with a sagittal angle of 56.39 ± 5.48° for the males and 59.47 ± 5.73° for the females, a lateral angle of 37.06 ± 2.56° for the males and 36.86 ± 3.27° for the females. When placing the screws, any excessive deflection may puncture the sacral or iliac cortex, damaging the tissues proximal to the pelvis catastrophically. Especially, excessive caudal insertion of screws is likely to violate the sciatic notch, which may lead to injury of the superior gluteal artery and nerve, both of which pass above the piriformis muscle [7]. With respect to the insertion points of S1AI screws, they were deeper from both the PSIS and the skin according to previous studies [13, 15], which means S1AI screws can be much lower profile and have lower incidence of implant prominence.

Proper identification of the anatomical landmarks related to the recommended entry points is one of the most important factors for a successful pelvic fixation with SAI screws. Dr. Mattei et al. [6] reported that to obtain an adequate lateral exposure for proper identification of the entry point for the S2AI screws, the skin incision and muscle dissection usually have to be extended distally to the level of the third or fourth sacral segment. However, this area is the portion of the wound most prone to breakdown. Any additional dissection of the skin, subcutaneous tissue, or muscle in this area increases the risk of blood loss and wound healing problems. Though have not been assessed biomechanically, the S1 alar iliac screw might achieve more structural stability than S1 pedicle screw for being placed more anteriorly and laterally, as McCord DH et al. [23] demonstrated. Thus, under some circumstances such as short lumbar-sacral fixation for osteoporotic patients, S2AI screw might not be necessarily needed taking wound healing into consideration while S1AI screw would be stable enough for fixation. More biomechanical data should be provided in further study.

It needs to be emphasized that this study was carried out in normal pelvis without any deformity. As lumbosacral pelvic fixation is also frequently carried out in deformity surgery, variability of the anatomical structures in deformity surgery would limit the application of such data in clinical practice. Therefore, in cases where there is pelvis asymmetry, planning of the screw trajectory and length have to be individualized, intraoperative fluoroscope and/or navigation systems may aid the surgeon to the final trajectory and screw size parameters [24].

## Conclusions

In summary, it is feasible for S1AI screws to be used in most of Chinese adult patients. Our findings indicated that the screw inserts at the lateral sacral crest between the S-1 foramen and the margin of the S-1 superior endplate through the sacroiliac joint and iliac wings with approximately 37° of lateral angulation in transverse plane and 56° for the males (59° for the females) of caudal angulation in the sagittal planes. However, more researches need to be conducted about SAI screws and spinopelvic fixation is still challenging.

## Abbreviations

3D: Three-dimension
Cortex. D.: The narrowest distance between the trajectory and the nearest inner cortex surface
CT: Computed tomography
Iliac W.: The narrowest iliac width between the inner cortices of the ilium in the transverse plane
Max. L.: The maximal length of the trajectory from the starting point to the anterior cortex
Mid. D.: The distance of the starting point lateral from the middle line of sacrum
PSIS D.: The distance from the starting point to the PSIS
S1AI: S1 alar iliac
S2AI: S2 alar iliac
Sac. L.: The length of intrasacral trajectory
Sag. A.: The caudal trajectory angulation in the sagittal plane
Skin D.: The shortest distance from the starting point to the skin
Tsv. A.: The lateral angulation in the transverse plane

## References

[1] Kostuik JP (2005). Spinopelvic fixation. Neurol India.

[2] Moshirfar A, Rand FF, Sponseller PD, Parazin SJ, Khanna AJ, Kebaish KM, Stinson JT, Riley LH (2005). Pelvic fixation in spine surgery. Historical overview, indications, biomechanical relevance, and current techniques. J Bone Joint Surg Am.

[3] Schwend RM, Waters PM, Hey LA, Hall JE, Emans JB (1992). Treatment of severe spondylolisthesis in children by reduction and L4-S4 posterior segmental hyperextension fixation. J Pediatr Orthop.

[4] Sponseller PD, Zimmerman RM, Ko PS, ter Gunne AFP, Mohamed AS, Chang T-L, Kebaish KM (2010). Low profile pelvic fixation with the sacral alar iliac technique in the pediatric population improves results at two-year minimum follow-up. Spine.

[5] Crawford CH, Carreon LY, Bridwell KH, Glassman SD (2012). Long fusions to the sacrum in elderly patients with spinal deformity. Eur Spine J.

[6] Mattei TA, Fassett DR (2013). Combined S-1 and S-2 sacral alar-iliac screws as a salvage technique for pelvic fixation after pseudarthrosis and lumbosacropelvic instability. J Neurosurg Spine.

[7] O’Brien JR, Yu WD, Bhatnagar R, Sponseller P, Kebaish KM (2009). An anatomic study of the S2 iliac technique for lumbopelvic screw placement. Spine.

[8] Martin CT, Witham TF, Kebaish KM (2011). Sacropelvic fixation: two case reports of a new percutaneous technique. Spine.

[9] Stevens DB, Beard C. Segmental spinal instrumentation for neuromuscular spinal deformity. Clin Orthop Relat Res. 1989;(242):164-8. 2706847

[10] Kim Y-Y, Ha K-Y, Kim S-I, Oh I-S (2015). A study of sacral anthropometry to determine S1 screw placement for spinal lumbosacral fixation in the Korean population. Eur Spine J.

[11] Kwan MK, Jeffry A, Chan CYW, Saw LB (2011). A radiological evaluation of the morphometry and safety of S1, S2 and S2-ilium screws in the Asian population using three dimensional computed tomography scan: an analysis of 180 pelvis. Surg Radiol Anat.

[12] Jain A, Hassanzadeh H, Strike SA, Menga EN, Sponseller PD, Kebaish KM (2015). Pelvic fixation in adult and pediatric spine surgery: historical perspective, indications, and techniques. J Bone Joint Surg Am.

[13] Chang T-L, Sponseller P, Kebaish K, Fishman E (2008). Low profile pelvic fixation anatomic parameters for sacral alar iliac fixation vs. traditional iliac fixation. Spine J.

[14] Kebaish KM (2010). Sacropelvic fixation. Spine.

[15] Zhu F, Bao HD, Yuan S, Wang B, Qiao J, Zhu ZZ, Liu Z, Ding YT, Qiu Y (2013). Posterior second sacral alar iliac screw insertion: anatomic study in a Chinese population. Eur Spine J.

[16] Mazur MD, Ravindra VM, Schmidt MH, Brodke DS, Lawrence BD, Riva-Cambrin J, Dailey AT (2015). Unplanned reoperation after lumbopelvic fixation with S-2 alar-iliac screws or iliac bolts. J Neurosurg Spine.

[17] JR OB, Yu W, Kaufman BE, Bucklen B, Salloum K, Khalil S, Gudipally M (2013). Biomechanical evaluation of S2 alar-iliac screws. Spine.

[18] Liu Z, Qiu Y, Yan H, Hu Z-s, Zhu F, Qiao J, Xu L-l, Wang B, Yu Y, Qian B-P, Zhu Z-z (2016). S2 alar-iliac fixation: a powerful procedure for the treatment of kyphoscoliosis. Orthop Surg.

[19] Shabtai L, Andras LM, Portman M, Harris LR, Choi PD, Tolo VT, Skaggs DL (2016) Sacral Alar Iliac (SAI) screws fail 75% less frequently than iliac screws in neuromuscular scoliosis. J Pediatr Orthop. 2017;37(8):e470-e475. 10.1097/BPO.0000000000000720. 10.1097/BPO.0000000000000720 26756987

[20] Park J-H, Hyun S-J, Kim K-J, Jahng T-A (2015). Free hand insertion technique of S2 sacral alar-iliac screws for spino-pelvic fixation: technical note, acadaveric study. J Korean Neurosurg Soc.

[21] Pham MH, Jakoi AM, Hsieh PC (2016). S-1 and S-2-alar-iliac screw fixation via intraoperative navigation. Neurosurg Focus.

[22] Camp JF, Caudle R, Ashmun RD, Roach J (1990). Immediate complications of Cotrel-Dubousset instrumentation to the sacro-pelvis. Clin Biomech Study Spine.

[23] McCord DH, Cunningham BW, Shono Y, Myers JJ, McAfee PC (1992). Biomechanical analysis of lumbosacral fixation. Spine.

[24] Hu X, Lieberman IH (2016) Robotic-guided sacro-pelvic fixation using S2 alar-iliac screws: feasibility and accuracy. Eur Spine J. 2017;26(3):720-25. 10.1007/s00586-016-4639-5. 10.1007/s00586-016-4639-5 27272491

